# Case report: anti-N-Methyl-D-Aspartate receptor encephalitis and bilateral temporal calcifications

**DOI:** 10.1186/s12883-020-01962-3

**Published:** 2020-10-23

**Authors:** Yujie Bu, Tinghua Zhang, Jia Guo

**Affiliations:** Department of Neurology, Lanzhou University Second Hospital, Lanzhou, 730030 Gansu China

**Keywords:** Anti-NMDAR encephalitis, Epilepsy, Bilateral temporal calcifications, CEC

## Abstract

**Background:**

In this study, we report a case of a young female who was hospitalized for seizures and diagnosed with anti–N-methyl-D-aspartate receptor (NMDAR) encephalitis.

**Case presentation:**

The main feature of this patient was bilateral temporal calcifications detected by routine head computed tomography (CT). The co-existence of anti-NMDAR encephalitis and cerebral calcifications has not been reported. We supposed that the patient had an incomplete form of celiac disease (CD), epilepsy and cerebral calcifications syndrome (CEC). The patient's symptoms were alleviated by a series of treatments, and she remained stable during the follow-ups.

**Conclusions:**

Our findings confirm the rarity co-existing anti-NMDAR encephalitis and cerebral calcifications. In future clinical work, we need to elucidate the relationship between anti-NMDAR encephalitis and cerebral calcifications, and the association between anti-NMDAR encephalitis and other co-existing autoimmune disorders.

## Background

Anti-NMDAR encephalitis is an immune-mediated disorder that was first reported in 2007 in patients with psychiatric symptoms, amnesia, seizures, abnormal movements, autonomic dysfunction, and decreased level of consciousness or hypoventilation [1]. Since then, a rapid expansion occurred in the number of anti-NMDAR encephalitis cases reported, with or without an associated tumor. Anti-NMDAR encephalitis is more frequent in epilepsy seizures than other types of autoimmune encephalitis [2]. We treated a female patient with anti-NMDAR encephalitis who was hospitalized for seizures. The CT of her brain showed bilateral temporal calcifications.

The association between epilepsy and cerebral calcifications has been extensively studied [3, 4]. In 1992, Gobbi et al. [5] first described the triad of CD, epilepsy, and posterior cerebral calcification and named it CEC in 2005 [6]. Based on the manifestations and imaging findings of our patient, we presumed that our patient suffered from an incomplete form of CEC. Following adherence to a strict gluten-free diet (GFD), along with prednisone and antiepileptic drug administration, the patient's symptoms were alleviated and remained stable during the follow-ups.

Importantly, anti-NMDAR encephalitis co-existing with cerebral calcifications has not been previously reported. Therefore, this case provides critical insights into the possibility that cerebral calcifications might be a nonspecific manifestation of NMDAR encephalitis or a disease co-existing with anti-NMDAR encephalitis.

## Case Presentation

**Fig. 1:**
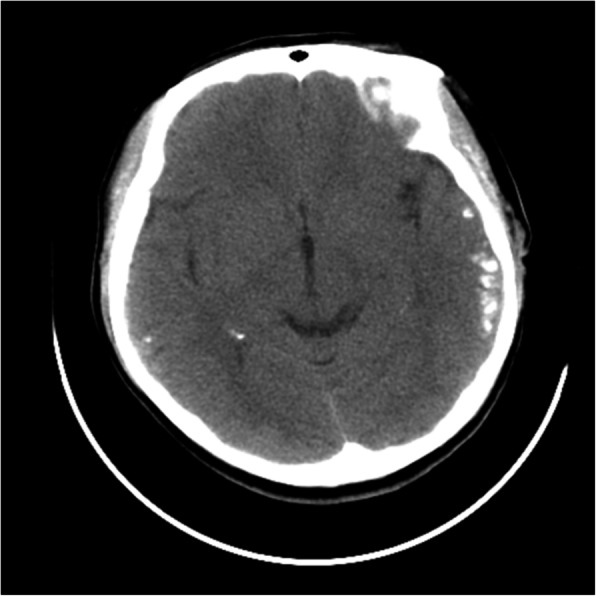
Plain computed tomography of the brain at presentation to the hospital showing patchy calcification in bilateral temporal cortico-subcortical areas (left > right)

A 25-year-old female nurse that worked in our department was admitted to hospital for a seizure. A CT scan of her brain showed bilateral temporal calcifications (Fig. 1). The patient had no prodromal features (history of infection or previous flu-like symptoms) and denied drug or alcohol ingestion. In our preceding daily work contacts, we had observed her strange personality and behavior. On general examination, we found no nevus flammeus on her face and supposed she had an atypical Sturge-Weber syndrome (SWS). Laboratory investigations showed that her complete blood count, renal and liver function, blood glucose, serum calcium, serum phosphorus, and parathyroid hormone were within the reference ranges. The screening tests for Hepatitis B, Hepatitis C, human immunodeficiency virus, and syphilis were negative. Her thyroid function test showed increases of anti-thyroid peroxidase antibodies (TPO-Ab) (> 1300 U/mL) and anti-thyroglobulin (209.60 U/mL), while fT3, fT4 and TSH were in normal rang. Ultrasound investigation revealed a swollen thyroid. The findings of the brain magnetic resonance imaging (MRI) findings were consistent with those of the head CT. 12-h video electroencephalogram revealed spikes and spike-slow waves discharged from left temporal electrodes with spread to the contralateral hemisphere during the sleep stage. She was administered antiepileptic medication (levetiracetam) which terminated her seizures, and she returned to work.

**Fig. 2:**
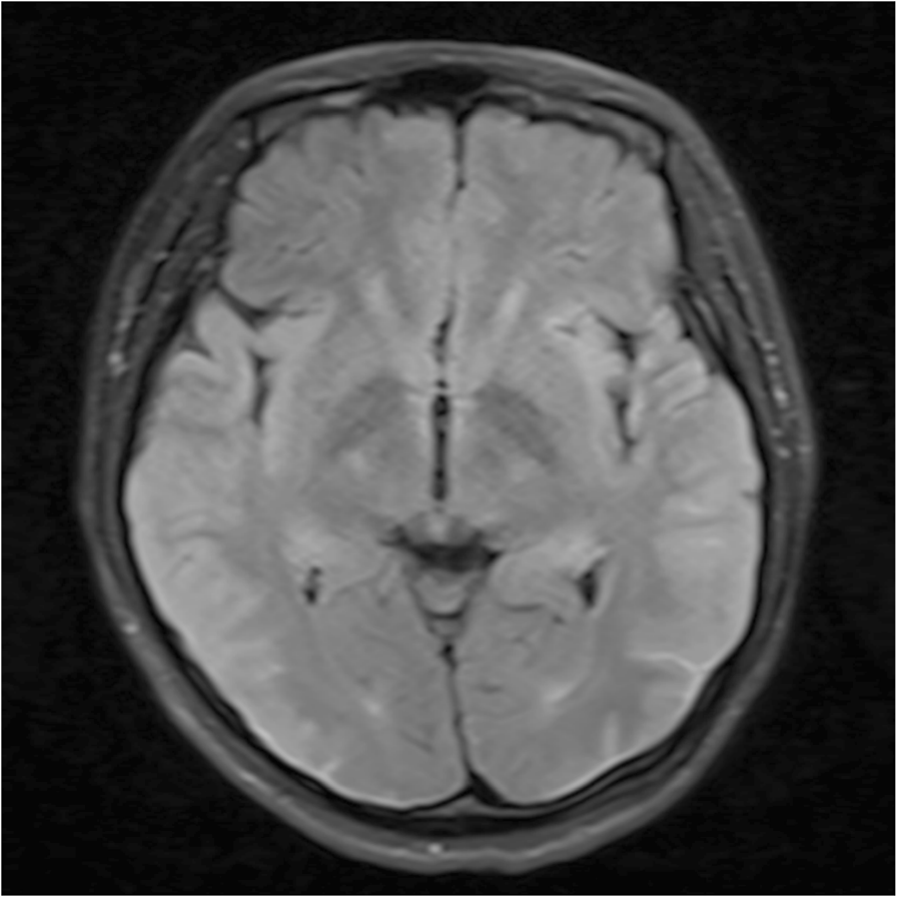
T2 fluid-attenuated inversion recovery (FLAIR) axial views showing obvious hyperintense signals and mild swilling in bilateral parietal-occipital lobe, temporal lobe and insular cortex

Eleven months later, the patient was hospitalized after a second seizure. Brain MRI showed slightly thickened and swollen bilateral parietal occipital lobe, temporal lobe, and insular cortexes (Fig. 2). The patient's strange personality and behavior and her two epileptic seizures were reminiscent of autoimmune encephalitis, although the MRI findings of autoimmune encephalitis are not specific, especially in anti-NMDAR encephalitis [7]. Therefore, a lumbar puncture was performed, and the results of cerebrospinal fluid (CSF) tests showed 11 WBCs/mm3, protein concentration of 0.56 g/L, normal glucose and chloride levels, Gram stain-negative. The serum was anti-NMDAR antibody-negative results, whereas the CSF was antibody-positive, established using cell-based assays (1:3.2, Euroimmun, Germany). Herpes simplex virus PCR and arbovirus antibody assays were not performed due to the low index of suspicion. The results we obtained confirmed her diagnosis of anti-NMDAR encephalitis [8]. Gynecological ultrasound was conducted with a normal result. 8-h video electroencephalogram monitoring did not show definite evidence of extreme delta brush, but detected several occasional spike-slow waves in each electrode, prominently in bilateral frontal and central-temporal electrodes. Due to the diagnosis of anti-NMDAR encephalitis, she underwent intravenous steroid therapy (methylprednisolone, 1000 mg/d), followed by oral prednisone. The antiepileptic drug was changed to lamotrigine, which is cheaper. Since our patient’s condition was mild, and she responded well to steroids, IV immunoglobulin or plasma exchange was not used, and no other immunosuppressive drugs were administered after her discharge. She refused to be subjected to PET-CT imaging assessment due to the high cost. We suggested that the patient should have a gynecological ultrasound examination every six months.

**Fig. 3:**
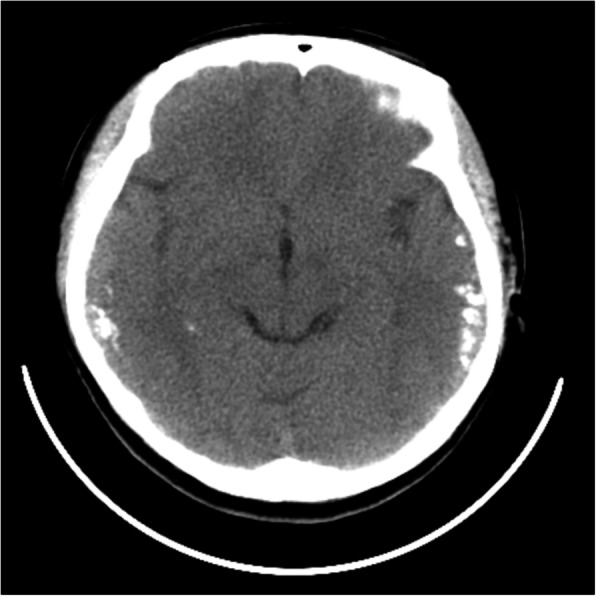
Plain computed tomography of the brain at third admission showing calcifications in bilateral temporal corticosubcortical areas were increased

Six months after the second admission, the patient’s condition remained stable. We found that her CSF antibody titers turned negative. However, two weeks later, her initial symptoms recurred. Laboratory screening results were normal but, the screening of CSF showed it was again anti-NMDAR antibody-positive (1:1, cell-based assays, Euroimmun, Germany). Additionally, mild growth of bilateral temporal calcifications was detected by CT (Fig. 3). The MRI of the brain showed that the cortical swelling had subsided significantly compared to the second admission. Gynecological ultrasound revealed the absence of a tumor. Serum folate levels were within the reference range. As aforementioned, cerebral calcifications were a significant specific feature of our patients. Many diseases, including CEC, can cause cerebral calcification and epilepsy. We reviewed the medical history of the patient in detail, including the abdominal symptoms. The patient reported that she had chronic diarrhea, of which her uncle and brother also had a history, and abdominal pain. To determine whether she had CD, we analyzed her serum for anti-autoantigen (tTG) antibodies (including antigliadin and antiendomysium antibodies), but the results were negative. She declined further investigation by duodenal endoscopic biopsy. We considered she was in a stable phase of CD, but still recommended that she had to adhere to a strict GFD. In the following ten months, the patient’s conditions gradually improved. The patient underwent chest and abdomen CT and pelvis ultrasound, no teratoma was found, and she returned to normal work and life activities.

## Discussion and Conclusion

In this report, we have described in detail a case with cerebral calcifications and seizures diagnosed with anti-NMDAR encephalitis. Cerebral calcifications and seizures are often associated with SWS. In the first episode in our patient, the symptoms of epilepsy and bilateral calcifications suggested a diagnosis of atypical SWS as previously reported due to the lack of nevus flammeus on her face. However, many other classic SWS features, such as mental retardation, hemiplegia/ hemiatrophy, and cerebral atrophy [9–11], were not present in this case, distinguishing her condition from SWS. The patient was tested and found to have a high TPO-Ab level. Commonly, the presence of Hashimoto's encephalopathy (HE) is to be considered in a patient with positive thyroid antibodies and seizures [12]. However, these diagnostic criteria for HE remain a diagnosis of exclusion since the antibodies are not specific to HE patients [13]. Antithyroid antibodies are non-specific and can co-exist with other autoimmune antibodies, such as the anti-NMDAR antibody [14]. Initially, we thought calcifications were the cause of her seizures, and the patient's symptoms were effectively controlled after receiving antiepileptic treatment.

At her second admission to hospital, the patient was diagnosed with anti-NMDAR encephalitis. Anti-NMDAR encephalitis is an autoimmune encephalitis [1] that can be simultaneously paraneoplastic- and autoimmune-mediated. These disorders are usually responsive to immunomodulatory therapies and/or removal of the associated tumors but can relapse [15, 16]. Here, the test results were initially antibody-positive in the CSF. During disease progression, the antibody-positive results were again obtained. Studies suggest that it is necessary to follow up patients’ antibodies in the serum and CSF after clinical recovery [15, 17]. We followed up antibody titers during the disease and established that the patient's antibody titer fluctuated largely. However, the degree of correlation between the serum and CSF antibody titers, relapses and outcomes, requires further larger and more robust investigation.

Our patient was hospitalized three times for epilepsy with significant bilateral temporal calcifications. Co-existence of anti-NMDAR encephalitis and cerebral calcifications has not been previously reported. Through literature retrieval, we found that epilepsy and intracranial calcification can occur simultaneously in CEC. In a review on CEC, 82% of cerebral calcifications were found to be located posteriorly. The remaining calcifications were either frontal, temporal, or (in a minority of patients) in the sub-cortical areas [18]. The intracranial calcifications located in the temporal lobe of our patient were relatively rare. Furthermore, the mechanism of cerebral calcifications is not entirely elucidated. Folate malabsorption, treatment with methotrexate and radiotherapy can induce cerebral calcifications similar to those in CEC [19], although none of these were related to the condition of our patient. Nevertheless, the underlying relationship of the triads has not been fully determined.

After repeatedly questioning the patient’s medical history, her mother recalled the patient had symptoms of chronic diarrhea, which her uncle and brother also experienced, and abdominal pains during childhood. To further clarify weather our patient had CD, we performed tests for anti-tTG antibodies with negative results. Previous studies have shown that the testing of anti-tTG antibodies in the serum is completely sensitive (100%) and reasonably specific (96%) [20]. However, a single negative result of serum antibodies cannot always rule out the possibility of CD for the rest of a patient's life. It is noteworthy that the three symptoms of CEC are not always simultaneously present and may appear at different ages [6]. One of the atypical forms of CEC is epilepsy and cerebral calcifications without CD [6]. We conjectured that our patient had an incomplete form of CEC. Studies have revealed that the early uptake of GFD has long-lasting protective and control effects on seizures [6, 18], whereas non-adherence to a GFD may lead to a progressive growth of cerebral calcifications [6]. Despite the administration of steroids and anti-seizure medication, our patient developed a third seizure. Then, after further adhering to a strict GFD, she was free of seizures and diarrhea for more than ten months. The improvement in the symptoms after the further GFD may indirectly indicate possible existence of CD. Therefore, patients with intracranial calcifications and anti-tTG antibodies should be followed-up for disease evaluation. In this case, further duodenal endoscopic biopsy was highly necessary.

Anti-NMDAR encephalitis is an autoimmune disorder. Similarly, CEC is an autoimmune disease that can affect different organ systems to varying extents [21]. The pathogeneses of these two conditions may be highly homologous, which is in agreement with previous reports in the literature of the co-existence of autoimmune diseases and anti-NMDAR encephalitis [22, 23]. The condition of our patient was complicated. We hereby present the first case report, in which anti-NMDAR encephalitis co-exists with bilateral temporal calcifications. This is also the first case in our work in which we suspected that intracranial calcifications were associated with CEC. The association between anti-NMDAR encephalitis and intracranial calcifications remains unclear. Therefore, further long-term patient follow-up is required to elucidate the association between anti-NMDAR encephalitis and bilateral temporal calcifications. Bilateral temporal calcifications can be a nonspecific manifestation of NMDAR encephalitis or of a disease co-existing with anti-NMDAR encephalitis.

## Data Availability

The datasets used and/or analysed during the current study are available from the corresponding author on reasonable request.

## Abbreviations

NMDAR: N-methyl-D-aspartate receptor
CT: Computed tomography
CD: Celiac disease
CEC: Cerebral calcifications syndrome
GFD: Gluten-free diet
SWS: Sturge-Weber syndrome
TPO-Ab: Thyroid peroxidase antibodies
MRI: Magnetic resonance imaging
CSF: Cerebrospinal fluid
